# Voluntary Medical Male Circumcisions for HIV Prevention — 13 Countries in Eastern and Southern Africa, 2017–2021

**DOI:** 10.15585/mmwr.mm7210a2

**Published:** 2023-03-10

**Authors:** Megan E. Peck, Katherine S. Ong, Todd Lucas, Pauline Harvey, Phenyo Lekone, Mpho Letebele, Vasavi T. Thomas, Vusi Maziya, Mandzisi Mkhontfo, Teruwork Gultie, Dejene Mulatu, Mesfin Shimelis, Tiruneh Zegeye, Ambrose W. Juma, Elijah Odoyo-June, Paul K. Musingila, John Njenga, Andrew Auld, Martin Kapito, Alice Maida, Wezi Msungama, Marcos Canda, Jotamo Come, Inacio Malimane, Mekondjo Aupokolo, Brigitte Zemburuka, Ida Kankindi, Samuel Malamba, Eric Remera, Emmanuel Tubane, Richard Machava, Nandi Maphothi, Peter Vranken, Mbaraka Amuri, Kokuhumbya J. Kazaura, Daimon Simbeye, Stella Alamo, Geoffrey Kabuye, Omega Chituwo, Royd Kamboyi, Joseph Masiye, John Mandisarisa, Sinokuthemba Xaba, Carlos Toledo

**Affiliations:** ^1^Divison of Global HIV and Tuberculosis, Center for Global Health, CDC; ^2^Division of Global HIV and Tuberculosis, CDC Botswana; ^3^Ministry of Health, Mbabane, Eswatini; ^4^Division of Global HIV and Tuberculosis, CDC Eswatini; ^5^ICAP, Addis Ababa, Ethiopia; ^6^Ministry of Health, Addis Ababa, Ethiopia; ^7^Division of Global HIV and Tuberculosis, CDC Ethiopia; ^8^National STD/AIDS Control Program, Ministry of Health, Nairobi, Kenya; ^9^Division of Global HIV and Tuberculosis, CDC Kenya; ^10^Division of Global HIV and Tuberculosis, CDC Malawi; ^11^Ministry of Health, Lilongwe, Malawi; ^12^Division of Global HIV and Tuberculosis, CDC Mozambique; ^13^Ministry of Health, Maputo, Mozambique; ^14^Ministry of Health and Social Services, Windhoek, Namibia; ^15^Division of Global HIV and Tuberculosis, CDC Namibia; ^16^Division of Global HIV and Tuberculosis, CDC Rwanda; ^17^Rwanda Biomedical Centre, Kigali, Rwanda; ^18^Ministry of Health, Kigali, Rwanda; ^19^Division of Global HIV and Tuberculosis, CDC South Africa; ^20^Division of Global HIV and Tuberculosis, CDC Tanzania; ^21^Division of Global HIV and Tuberculosis, CDC Uganda; ^22^Division of Global HIV and Tuberculosis, CDC Zambia; ^23^Ministry of Health, Lusaka, Zambia; ^24^Division of Global HIV and Tuberculosis, CDC Zimbabwe; ^25^Ministry of Health and Child Care, Harare, Zimbabwe.

In 2007, voluntary medical male circumcision (VMMC) was endorsed by the World Health Organization (WHO) and the Joint United Nations Programme on HIV/AIDS after it was found to be associated with approximately a 60% reduction in the risk for female-to-male transmission of HIV (*1*). As a result of this endorsement, the U.S. President’s Emergency Plan for AIDS Relief (PEPFAR), through partnerships with U.S. government agencies, including CDC, the U.S. Department of Defense, and the U.S. Agency for International Development, started supporting VMMCs performed in prioritized countries in southern and eastern Africa. During 2010–2016, CDC supported 5,880,372 VMMCs in 12 countries (*2*,*3*). During 2017–2021, CDC supported 8,497,297 VMMCs performed in 13 countries. In 2020, the number of VMMCs performed declined 31.8% compared with the number in 2019, primarily because of COVID-19–related disruptions to VMMC service delivery. PEPFAR 2017–2021 Monitoring, Evaluation, and Reporting data were used to provide an update and describe CDC’s contribution to the scale-up of the VMMC program, which is important to meeting the 2025 Joint United Nations Programme on HIV/AIDS (UNAIDS) target of 90% of males aged 15–59 years having access to VMMC services in prioritized countries to help end the AIDS epidemic by 2030 (*4*).

VMMC programs are incorporated into national HIV prevention portfolios. Services include voluntary HIV testing, HIV risk reduction education, screening and treatment of sexually transmitted infections, and linkage to care and treatment for clients who receive a positive HIV test result. During 2017–2021, CDC supported VMMC programs in 13 countries: Botswana, Eswatini, Ethiopia, Kenya, Malawi, Mozambique, Namibia, Rwanda, South Africa, Tanzania, Uganda, Zambia, and Zimbabwe. Not all of these countries were supported by CDC during the entire 5-year period.*

VMMC programs report indicators to the PEPFAR Monitoring, Evaluation, and Reporting database every quarter in accordance with the U.S. government fiscal year† (*5*). This analysis includes the annual number of CDC-supported VMMCs performed, as well as the following indicators: client age group, HIV test results among males who underwent testing at VMMC sites, attendance at postoperative follow-up visits within 14 days, and type of circumcision method (use of a WHO pre-qualified circumcision device as method of circumcision or traditional surgical technique). Age was categorized as <15 years, 15–29 years, and ≥30 years. The prevalence of HIV-positivity was calculated by dividing the number of males who received a positive test result for HIV by the number of males who underwent HIV testing at VMMC sites. All statistical analyses were performed using Stata software (version 16; StataCorp). This activity was reviewed by CDC and was conducted consistent with applicable federal law and CDC policy.§

During 2017–2021, CDC supported 8,497,297 VMMCs in 13 countries (Table) (Figure). During 2017–2019, the number of CDC-supported VMMCs increased annually, with a mean annual increase of 13.5%. During 2020, at the start of the COVID-19 pandemic, the number of VMMCs declined 31.8%, from 2,120,797 in 2019 to 1,447,147, with further reductions during 2021. During 2017–2019, 43.8% of all VMMCs were performed in clients aged 10–14 years and 45.7% in clients aged 15–29 years; the proportion performed in clients aged 15–29 years increased to 61.0% during 2020 and to 86.6% during 2021. Most (74.2%) VMMC clients participated in HIV testing services at VMMC sites; this proportion declined from 86.4% in 2017 to 48.7% in 2021.¶ Among the 5,595,239 males who underwent testing for HIV at VMMC sites, 44,745 (0.8%) received a positive result. HIV-positivity ranged from 0.4% in 2020 to 1.2% in 2021.

**TABLE:** CDC-supported voluntary medical male circumcisions — 13 countries in eastern and southern Africa, 2017–2021

Country	Fiscal year*	No. of VMMCs performed	Annual target achieved, %	Client age group, yrs, no. (%)			No. (%)			
				<15	15–29	≥30	VMMCs performed using a device^†^	Clients who received HIV testing at a VMMC site	Clients with a positive HIV test result^§^	Clients with postoperative follow-up within 14 days of VMMC
Botswana	2017	17,870	117.0	11,205 (62.7)	4,814 (26.9)	1,851 (10.4)	287 (1.6)	9,311 (52.1)	14 (0.2)	10,968 (61.4)
	2018	15,874	76.0	9,386 (59.1)	4,701 (29.6)	1,787 (11.3)	0 (—)	7,730 (48.7)	29 (0.4)	14,674 (92.4)
	2019	16,461	78.4	7,710 (46.8)	6,127 (37.2)	2,624 (15.9)	0 (—)	9,436 (57.3)	11 (0.1)	16,155 (98.1)
	2020^¶^	5,845	28.0	2,313 (39.7)	2,252 (38.6)	1,265 (21.7)	0 (—)	3,913 (66.9)	20 (0.5)	5,514 (94.3)
	2021	2,748	31.0	0 (—)**	1,539 (35.9)	2,748 (64.1)	0 (—)	1,437 (52.3)	25 (1.7)	2,720 (99.0)
	**Total**	**58,798**	**67.4**	**30,614 (50.8)**	**19,433 (32.2)**	**10,275 (17.0)**	**287 (0.5)**	**31,827 (54.1)**	**99 (0.3)**	**50,031 (85.1)**
Eswatini	2017^††^	0	0	0 (—)	0 (—)	0 (—)	0 (—)	0 (—)	0 (—)	0 (—)
	2018^††^	0	0	0 (—)	0 (—)	0 (—)	0 (—)	0 (—)	0 (—)	0 (—)
	2019	761	20.0	298 (39.2)	410 (53.9)	53 (7.0)	0 (—)	707 (92.9)	7 (1.0)	761 (100.0)
	2020^¶^	4,626	24.0	1,923 (41.6)	2,197 (47.5)	506 (10.9)	0 (—)	4,245 (91.8)	56 (1.3)	4,322 (93.4)
	2021	3,269	79.2	3 (0.1)**	2,561 (78.3)	705 (21.6)	0 (—)	2,984 (91.3)	6 (0.2)	3,259 (99.7)
	**Total**	**8,656**	**31.5**	**2,224 (25.7)**	**5,168 (59.7)**	**1,264 (14.6)**	**0 (—)**	**7,936 (91.7)**	**69 (0.9)**	**8,342 (96.4)**
Ethiopia	2017	10,910	96.2	5,290 (48.5)	4,610 (42.3)	1,010 (9.3)	0 (—)	8,149 (74.7)	3 (0.0)	10,905 (99.9)
	2018	20,302	112.0	9,023 (44.4)	9,312 (45.9)	1,967 (9.7)	0 (—)	13,941 (68.7)	43 (0.3)	19,874 (97.9)
	2019	23,776	98.3	10,024 (42.2)	11,768 (49.5)	1,984 (8.3)	0 (—)	21,907 (92.1)	7 (0.0)	23,169 (97.4)
	2020^¶^	33,483	82.1	3,198 (9.6)	25,681 (76.7)	4,604 (13.8)	0 (—)	31,189 (93.1)	30 (0.1)	32,540 (97.2)
	2021	45,499	101.1	0 (—)**	39,148 (86.0)	6,351 (14.0)	0 (—)	23,417 (51.5)	14 (0.1)	44,636 (98.1)
	**Total**	**133,970**	**96.1**	**27,535 (20.6)**	**90,519 (67.6)**	**15,916 (11.9)**	**0 (—)**	**98,603 (73.6)**	**97 (0.1)**	**131,124 (97.9)**
Kenya	2017	149,286	90.7	106,754 (71.5)	38,858 (26.0)	3,674 (2.5)	1,446 (1.0)	146,157 (97.9)	285 (0.2)	121,855 (81.6)
	2018	191,111	96.5	158,642 (83.0)	29,540 (15.5)	2,929 (1.5)	2,140 (1.1)	82,772 (43.3)	281 (0.3)	159,537 (83.5)
	2019	185,145	93.9	161,990 (87.5)	20,405 (11.0)	2,750 (1.5)	2,820 (1.5)	24,346 (13.1)	64 (0.3)	170,353 (92.0)
	2020^¶^	68,173	48.8	45,030 (66.1)	21,366 (31.4)	1,708 (2.5)	1,750 (2.6)	14,823 (21.7)	25 (0.2)	60,432 (88.6)
	2021	25,351	115.6	1 (0)**	22,803 (89.9)	2,547 (10.0)	2,789 (11.0)	7,671 (30.3)	16 (0.2)	24,683 (97.4)
	**Total**	**619,066**	**85.8**	**472,417 (79.2)**	**110,169 (18.5)**	**13,608 (2.3)**	**10,945 (1.8)**	**275,769 (44.5)**	**671 (0.2)**	**536,860 (86.7)**
Malawi	2017	30,136	86.1	5,612 (18.6)	21,455 (71.2)	3,069 (10.2)	0 (—)	30,063 (99.8)	104 (0.3)	24,219 (80.4)
	2018	46,004	92.0	4,199 (9.1)	37,562 (81.6)	4,243 (9.2)	109 (0.2)	45,780 (99.5)	520 (1.1)	37,216 (80.9)
	2019	52,062	104.1	3,205 (6.2)	45,015 (86.5)	3,842 (7.4)	824 (1.6)	51,791 (99.5)	434 (0.8)	52,041 (99.9)
	2020^¶^	34,239	38.9	4,423 (12.9)	27,677 (80.8)	2,139 (6.2)	371 (1.1)	28,482 (83.2)	14 (0.0)	34,239 (99.9)
	2021	70,178	97.5	0 (—)**	65,226 (92.9)	4,952 (7.1)	5,667 (8.1)	23,429 (33.4)	25 (0.1)	69,961 (99.7)
	**Total**	**232,619**	**78.8**	**17,439 (8.1)**	**196,935 (91.9)**	**18,245 (8.5)**	**6,971 (3.0)**	**179,545 (77.2)**	**1,097 (0.6)**	**217,676 (93.6)**
Mozambique	2017	189,225	62.5	96,218 (50.8)	83,211 (44.0)	9,796 (5.2)	0 (—)	178,615 (94.4)	4,350 (2.4)	144,708 (76.5)
	2018	233,069	90.9	131,881 (56.6)	90,365 (38.8)	10,823 (4.6)	0 (—)	219,906 (94.4)	4,530 (2.1)	200,060 (85.8)
	2019	222,887	83.1	130,731 (58.7)	82,253 (36.9)	9,903 (4.4)	0 (—)	206,983 (92.9)	4,736 (2.3)	193,267 (86.7)
	2020^¶^	120,464	42.9	59,232 (49.2)	54,596 (45.3)	6,636 (5.5)	0 (—)	57,490 (47.7)	1,109 (1.9)	98,738 (82.0)
	2021	46,292	84.3	0 (—)**	37,873 (81.8)	8,419 (18.2)	0 (—)	31,419 (67.9)	3,183 (10.1)	39,549 (85.4)
	**Total**	**811,937**	**69.8**	**418,062 (51.5)**	**348,298 (42.9)**	**45,577 (5.6)**	**0 (—)**	**694,413 (85.5)**	**17,908 (2.6)**	**676,322 (83.3)**
Namibia	2017	15,579	70.1	5,037 (33.0)	7,937 (51.9)	2,305 (15.0)	0 (—)	9,377 (60.2)	63 (0.7)	15,106 (97.0))
	2018	19,384	82.7	8,807 (46.0)	8,393 (43.8)	1,957 (10.2)	0 (—)	9,752 (50.3)	36 (0.4)	18,857 (97.3)
	2019	17,059	73.3	7,480 (45.5)	7,235 (44.0)	1,711 (10.4)	546 (3.2)	8,829 (51.8)	44 (0.5)	15,614 (91.5)
	2020^¶,††^	0	0	0 (—)	0 (—)	0 (—)	0 (—)	0 (—)	0 (—)	0 (—)
	2021^††^	0	0	0 (—)	0 (—)	0 (—)	0 (—)	0 (—)	0 (—)	0 (—)
	**Total**	**52,022**	**75.4**	**21,324 (41.9)**	**23,565 (46.3)**	**5,973 (11.7)**	**546 (1.0)**	**27,958 (53.7)**	**143 (0.5)**	**49,577 (95.3)**
Rwanda	2017	91,689	191.2	25,123 (27.4)	63,301 (69.1)	3,245 (3.5)	53,351 (58.2)	90,564 (98.8)	281 (0.3)	91,662 (99.9)
	2018	75,338	222.2	28,866 (38.3)	43,323 (57.5)	3,149 (4.2)	30,178 (40.1)	68,384 (90.8)	22 (0.0)	75,201 (99.8)
	2019	79,622	152.6	23,933 (30.1)	52,202 (65.6)	3,487 (4.4)	15,167 (19.0)	44,729 (56.2)	14 (0.0)	79,420 (99.7)
	2020^¶^	140,984	143.2	35,383 (25.1)	100,287 (71.1)	5,314 (3.8)	7,615 (5.4)	0 (—)^§§^	0 (—)^§§^	140,784 (99.9)
	2021	181,539	156.4	14 (0)**	163,800 (90.2)	17,725 (9.8)	2,016 (1.1)	0 (—)^§§^	0 (—)^§§^	179,435 (98.9)
	**Total**	**569,172**	**163.3**	**113,319 (19.9)**	**422,916 (74.3)**	**32,920 (5.8)**	**108,327 (19.0)**	**203,677 (35.8)**	**317 (0.2)**	**566,502 (99.5)**
South Africa	2017	232,198	94.3	91,312 (39.3)	114,436 (49.3)	26,450 (11.4)	886 (0.4)	140,960 (60.7)	4,390 (3.1)	169,955 (73.2)
	2018	284,202	81.7	144,208 (50.8)	107,826 (38.0)	32,037 (11.3)	0 (—)	260,025 (91.5)	4,524 (1.7)	189,787 (66.8)
	2019	332,096	109.1	125,598 (38.0)	175,228 (53.0)	30,055 (9.1)	0 (—)	285,267 (85.9)	1,795 (0.6)	247,819 (74.6)
	2020^¶^	144,622	46.4	34,347 (23.7)	89,195 (61.7)	21,080 (14.6)	0 (—)	134,101 (92.7)	412 (0.3)	131,951 (91.2)
	2021	164,995	52.4	0 (—)**	113,625 (68.9)	51,369 (31.1)	0 (—)	142,756 (86.5)	1,068 (0.7)	152,267 (92.3)
	**Total**	**1,158,113**	**75.9**	**395,465 (34.2)**	**600,310 (51.9)**	**160,991 (13.9)**	**886 (0.1)**	**1,022,493 (88.3)**	**14,189 (1.4)**	**891,779 (77.0)**
Tanzania	2017	290,041	91.7	131,039 (45.2)	136,000 (46.9)	23,002 (7.9)	0 (—)	222,693 (76.8)	547 (0.2)	258,342 (89.1)
	2018	451,073	92.8	206,288 (45.7)	209,371 (46.4)	35,414 (7.9)	0 (—)	450,318 (99.8)	674 (0.1)	390,295 (86.5)
	2019	453,764	110.4	193,883 (42.7)	223,742 (49.3)	36,139 (8.0)	1,517 (0.3)	59,909 (13.2)	105 (0.2)	438,954 (96.7)
	2020^¶^	299,967	104.4	140,485 (46.8)	140,580 (46.9)	18,902 (6.3)	817 (0.3)	0 (—)^§§^	0 (—)^§§^	296,017 (98.7)
	2021	337,989	95.9	0 (—)**	304,264 (90.0)	33,725 (10.0)	0 (—)	0 (—)^§§^	0 (—)^§§^	334,933 (99.1)
	**Total**	**1,832,834**	**98.9**	**671,695 (39.3)**	**891,557 (52.1)**	**147,182 (8.6)**	**2,334 (0.1)**	**732,920 (40.0)**	**1,326 (0.2)**	**1,718,541 (93.8)**
Uganda	2017	334,515	71.5	68,104 (33.2)	119,705 (58.4)	17,175 (8.4)	1,590 (0.5)	310,211 (92.7)	1,324 (0.4)	296,092 (88.5)
	2018	340,168	100.1	144,585 (49.4)	128,088 (43.8)	20,029 (6.8)	134 (0.0)	319,255 (93.9)	5,422 (1.7)	321,776 (94.6)
	2019	336,947	98.1	140,769 (41.8)	168,459 (50.0)	27,603 (8.2)	399 (0.1)	283,062 (84.0)	612 (0.2)	321,085 (95.3)
	2020^¶^	291,955	73.2	65,981 (22.6)	193,944 (66.4)	32,030 (11.0)	925 (0.3)	227,315 (77.9)	497 (0.2)	284,636 (97.5)
	2021	153,534	103.0	544 (0.4)	132,983 (86.6)	20,007 (13.0)	7,627 (5.0)	89,554 (58.3)	284 (0.3)	143,644 (93.6)
	**Total**	**1,457,119**	**85.8**	**419,983 (31.0)**	**743,179 (54.9)**	**116,844 (8.6)**	**10,675 (0.7)**	**1,229,397 (84.4)**	**8,139 (0.7)**	**1,367,233 (93.8)**
Zambia	2017	181,767	171.4	68,397 (37.6)	97,113 (53.4)	16,237 (8.9)	477 (0.3)	173,555 (95.5)	824 (0.5)	175,361 (96.5)
	2018	173,425	128.2	48,704 (28.1)	109,385 (63.1)	15,328 (8.8)	391 (0.2)	170,722 (98.4)	482 (0.3)	162,355 (94.0)
	2019	271,099	167.7	67,514 (24.9)	176,690 (65.2)	26,852 (9.9)	2,371 (0.9)	226,737 (83.6)	570 (0.3)	259,892 (95.9)
	2020^¶^	240,857	126.9	31,032 (12.9)	188,853 (78.4)	20,972 (8.7)	4,738 (2.0)	66,969 (27.8)	91 (0.1)	233,739 (97.0)
	2021	282,259	139.0	0 (—)**	258,048 (91.4)	24,211 (8.6)	12,217 (4.3)	39,859 (14.1)	71 (0.2)	276,061 (97.8)
	**Total**	**1,149,407**	**144.4**	**215,647 (18.8)**	**830,089 (72.2)**	**103,600 (9.0)**	**20,194 (1.8)**	**677,842 (59.0)**	**2,038 (0.3)**	**1,107,408 (96.3)**
Zimbabwe	2017	103,677	103.7	43,383 (41.9)	51,357 (49.5)	8,914 (8.6)	5,037 (4.9)	103,546 (99.9)	270 (0.3)	99,821 (96.3)
	2018	70,494	66.7	24,026 (34.1)	39,383 (55.9)	7,083 (10.0)	0 (—)	70,454 (99.9)	111 (0.2)	64,721 (91.8)
	2019	129,118	102.7	42,994 (33.3)	72,084 (55.9)	13,966 (10.8)	52 (0.0)	129,044 (99.9)	119 (0.1)	124,692 (96.6)
	2020^¶^	61,932	48.0	20,059 (32.4)	35,674 (57.6)	6,199 (10.0)	0 (—)	61,880 (99.9)	86 (0.1)	59,796 (96.6)
	2021	48,363	37.1	0 (—)**	39,042 (80.7)	9,321 (19.3)	221 (0.5)	47,935 (99.1)	73 (0.2)	45,513 (94.1)
	**Total**	**413,584**	**70.0**	**130,462 (31.6)**	**237,540 (57.4)**	**45,483 (11.0)**	**5,310 (1.3)**	**412,859 (99.8)**	**659 (0.2)**	**394,543 (95.4)**
All countries	2017	1,646,893	89.7	657,474 (43.3)	742,797 (49.0)	116,728 (7.7)	63,074 (3.8)	1,423,201 (86.4)	12,455 (0.9)	1,418,994 (86.2)
	2018	1,920,444	95.3	918,615 (49.1)	817,249 (43.6)	136,746 (7.3)	32,952 (1.7)	1,719,039 (89.5)	16,674 (1.0)	1,654,353 (86.1)
	2019	2,120,797	106.8	916,129 (43.2)	1,041,618 (49.2)	160,969 (7.6)	23,696 (1.1)	1,352,040 (63.8)	8,511 (0.6)	1,942,461 (91.6)
	2020^¶^	1,447,147	72.2	443,406 (30.6)	882,302 (61.0)	121,355 (8.4)	16,216 (1.1)	630,407 (62.7)^§§^	2,340 (0.4)	1,382,708 (95.5)
	2021	1,362,016	92.5	562 (0)	1,180,912 (86.6)	182,080 (13.4)	30,537 (2.2)	410,461 (48.7)^§§^	4,765 (1.2)	1,316,661 (96.7)
	**Total**	**8,497,297**	**91.2**	**2,936,186 (35.3)**	**4,664,878 (56.1)**	**717,082 (8.6)**	**166,475 (2.0)**	**5,595,239 (74.2)** ^ §§^	**44,745 (0.8)**	**7,715,177 (90.8)**

**Abbreviations:** PEPFAR = U.S. President’s Emergency Plan for AIDS Relief; VMMC = voluntary medical male circumcision.

* October 1–September 30.

^†^ VMMCs performed using a device refers to use of World Health Organization–prequalified circumcision device as method of circumcision instead of traditional surgical technique.

^§^ HIV prevalence was calculated by dividing the number of males who received positive HIV test results by the number undergoing HIV testing services at VMMC sites.

**^¶^** COVID-19 was declared a pandemic in 2020.

** In 2021 most countries reported zero or a small number of VMMCs conducted in clients aged <15 years because of a change in VMMC age eligibility to 15 years with the exception of the ShangRing device (Wuhu Snnda Medical Treatment Appliance Technology).

^††^ Eswatini in 2017 and 2018 and Namibia in 2020 and 2021 did not have a CDC-supported VMMC program.

^§§^ Rwanda and Tanzania stopped conducting PEPFAR-supported HIV testing at VMMC sites in 2020 and 2021. These countries were excluded from the combined country estimates.

**FIGURE:**
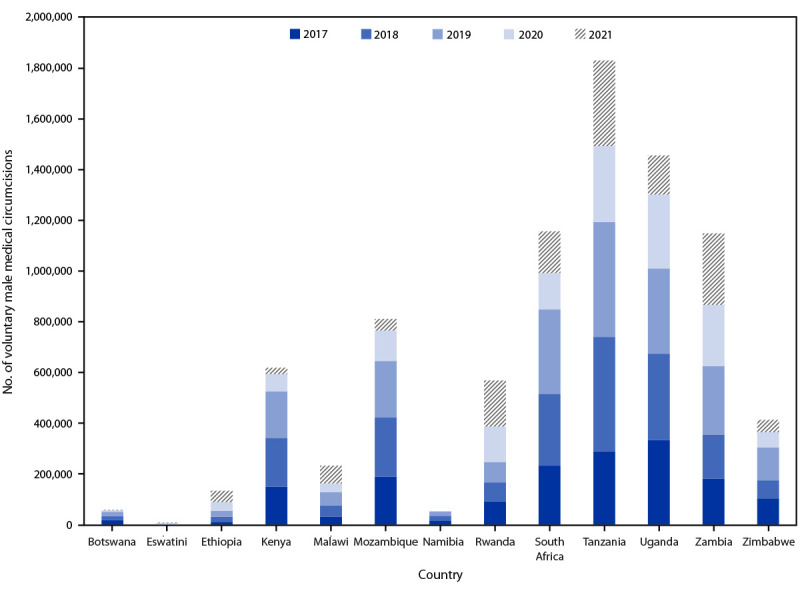
CDC-supported voluntary medical male circumcisions, by year — 13 eastern and southern African countries, 2017–2021* * COVID-19 was declared a pandemic in 2020.

Among all VMMC clients during 2017–2021, 90.8% returned for a follow-up visit within 14 days. Postoperative follow-up visits increased among all countries from, 86.2% in 2017 to 96.7% in 2021. Two percent (166,475) of all circumcisions were performed using a device; this proportion was highest (3.8%) in 2017, declined annually until 2020, then increased from 1.1% in 2020 to 2.2% in 2021.

## Discussion

Overall, substantial progress has been made in scaling up CDC-supported VMMC programs, with 8,497,297 VMMCs performed during 2017–2021. Increased programmatic experience in VMMC scale-up and the continued prioritization of VMMC by ministries of health and global stakeholders have contributed to this progress. The decreased number of VMMCs performed during 2020 was largely related to mitigation measures implemented to prevent the spread of COVID-19. In addition, in 2020, based on 2015–2018 data showing that certain severe adverse events associated with VMMC, while very rare, were higher among clients aged 10–14 years (2.9 per 100,000 procedures) than among clients aged ≥15 years (1.6 per 100,000 procedures), PEPFAR increased the age eligibility for VMMC to ≥15 years (*6*). This change in age eligibility likely contributed to the increase in the proportion of VMMCs performed in persons aged 15–29 years during 2020 and 2021.

During 2017–2021, a total of 44,745 males who underwent testing at a VMMC site received a positive HIV test result. VMMC sites serve as an important entry point for HIV testing; without this opportunity, many cases of HIV infection among males might go undiagnosed. The decrease in HIV testing at VMMC sites in 2020 and 2021 was likely due to changes in testing approaches after the 2019 PEPFAR recommendations to provide targeted testing including screening for clients at higher risk for HIV (*7*). VMMC postoperative follow-up visits increased in all countries during 2017–2021. The percentage of males who returned for a postoperative follow-up visit within 14 days exceeded the recommended 80%, which helped to facilitate timely detection of an adverse event. To reduce the transmission of COVID-19, many countries conducted virtual follow-up visits during 2020. In 2021, PEPFAR supported this approach based on evidence from the scientific literature and programmatic success (*8*).

VMMC programs can use WHO-prequalified male circumcision devices which typically includes application of a device for removal of the foreskin, as an alternative to traditional surgical circumcision techniques. The overwhelming majority of VMMCs are still performed through conventional surgical methods; the decision to introduce a circumcision device is country-specific, with many programs still piloting use of the devices. Device-based circumcisions declined from 3.8% during 2017 to 1.1% during 2019 and 2020; one factor contributing to the decline might be the lack of programmatic experience to scale up use of the ShangRing device (Wuhu Snnda Medical Treatment Appliance Technology) when practitioners started to phase out the PrePex device (Circ MedTech, Ltd.) during 2016–2018 after reports of tetanus in patients who received VMMC with this device (*9*). During 2021, more programs scaled up device-based circumcisions, and their use increased to 2.2% from 1.1% during 2020. 

The findings in this report are subject to at least three limitations. First, only CDC-supported VMMC results are reported, so the actual number of VMMCs performed might be higher than that reported here. Second, Monitoring, Evaluation, and Reporting data are subject to reporting and data entry errors. Finally, the data used for this analysis cannot be used to directly assess progress towards reaching the goal of 90% of eligible males having access to VMMC services. 

Modeling analyses have estimated that the 26.8 million PEPFAR-supported VMMCs performed during 2008–2019 in prioritized countries have helped prevent 340,000 new HIV infections; this estimate is projected to increase to 1.8 million by 2030, given that VMMC provides a lifelong reduction in HIV risk (*10*). CDC’s continued support of the VMMC program is a critical component of ending the AIDS epidemic and reaching the UNAIDS 2025 target of 90% of eligible males having access to VMMC in prioritized countries (*4*). Prioritization of uncircumcised males living in areas of high HIV incidence and those at highest risk for HIV can maximize VMMC’s contribution to HIV epidemic control.

**Summary**

**What is already known about this topic?**

Voluntary medical male circumcision (VMMC) is associated with an approximately 60% reduction in the risk for female-to-male transmission of HIV. The U.S. President’s Emergency Plan for AIDS Relief, through CDC and other organizations, has supported VMMC for HIV prevention in eastern and southern Africa. During 2010–2016, CDC supported 5,880,372 VMMCs performed in 12 countries.

**What is added by this report?**

During 2017–2021, CDC supported an additional 8,497,297 VMMCs performed in 13 countries in eastern and southern Africa. Compliance with postoperative follow-up visits within 14 days of VMMC was high, and use of device-based circumcisions remains low.

**What are the implications for public health practice?**

CDC’s continued support of the VMMC program is a critical component to ending the AIDS epidemic and reaching the Joint United Nations Programme on HIV/AIDS 2025 target of 90% of eligible males having access to VMMC in prioritized countries.

## Footnotes

* Eswatini started receiving CDC support in 2019, and in 2020 Namibia shifted PEFPAR support from CDC to another U.S. government agency.

† Years in VMMC program data refer to U.S. government fiscal years (October 1–September 30).

§ 45 C.F.R. part 46, 21 C.F.R. part 56; 42 U.S.C. Sect. 241(d); 5 U.S.C. Sect. 552a; 44 U.S.C. Sect. 3501 et seq.

¶ Rwanda and Tanzania stopped reporting HIV testing rates in fiscal year 2020 because of a change in funding and testing practices at VMMC sites.
